# Needs and Challenges to Improve Rehabilitative Care for Parents with Substance Use Disorder - A Qualitative Study from the Expert’s Perspective

**DOI:** 10.2147/SAR.S547025

**Published:** 2025-10-17

**Authors:** Laura Hoffmann, Ananda Stullich, Johannes Stephan, Jan Gehrmann, Matthias Richter

**Affiliations:** 1 Department Health and Sport Sciences, TUM School of Medicine and Health, Technical University of Munich, Munich, Germany; 2 Department Clinical Medicine, TUM School of Medicine and Health, Technical University of Munich, Munich, Germany

**Keywords:** addiction, family, children, rehabilitation, drugs, inpatient treatment

## Abstract

**Purpose:**

About 3 million children have lived in recent years with at least one parent who is addicted to legal and/or illegal substances in Germany. Parental substance use disorder represents a considerable social and economic burden for society. It also has severe health consequences for the parents themselves as well as for their children.

The topic of parenthood and the needs of parents with substance use disorders have so far received little attention in inpatient rehabilitative treatment in Germany. Therefore, the study aims to explore the parenting-related needs of parents with substance use disorders in inpatient rehabilitation treatment from an expert perspective. We also aim to uncover structural and general challenges in relation to the topic of “parenting and substance use disorder”.

**Patients and Methods:**

Semi-structured expert interviews were conducted at different intervention and non-intervention clinics. The data analysis was based on structured content analysis.

**Results:**

The results reveal a strong need for rehabilitation measures that take the topic of parenthood into account. This would include, for example, different parenting-related offers such as organizing daily life, shaping the relationship with the children, programs about the general development of children, expressing and regulating emotions, and assistance with official/administrative matters. Further, there are various structural issues that need to be addressed.

**Conclusion:**

Our results emphasize the concrete need for an evidence-based intervention for family-focused addiction treatment. In this way, care and the resulting health, quality of life, and functionality of the affected families can be improved in the future.

## Introduction

### Substance Use Disorder in Parents and Affected Children

International data on children who live with parents who use (il)legal substances or have a substance use disorder (SUD) is rare. So far, only estimates are available. Estimates showed that more than 21 million children lived with a parent who uses illicit substances in the United States (US). More than two million children lived with a parent who had an illicit SUD.^1^ Combined US data from 2009 to 2014 illustrate that 8.7 million children aged 17 or younger (12.3%) lived in households with at least one parent affected by SUD, not limited to illegal substances.^2^ Estimates for Europe are also rarely available. The only numbers that are available concern parents entering treatment, but it needs to be acknowledged that this population is only a partial representation of all persons using (il)legal substances living with children in Europe.^3^ In 2022, around 111.000 users of cocaine, opioids, cannabis, and amphetamine-stimulants entered the treatment system in Europe for the first time.^4^ The number of affected people who have already used treatment multiple times is even higher. Data on those entering treatment for “drug use problems” in 26 European countries showed that about 10% of the clients lived with children.^3^

In Germany, around 8.2 million people are addicted to (il)legal substances or gambling; almost 10 million relatives are affected, including a vast number of children.^5^ Reliable data on the exact number of affected children living with at least one parent with SUD is not available, even though different studies have tried to measure the number of children living with one or two parents with SUD.^6^ A direct comparison of the results is not possible, as different definitions of parental addiction were used (dependence vs abuse vs risky consumption vs binge drinking), and different survey methods were applied. However, the stronger the orientation towards the official criteria of SUD, the lower the number of cases. Studies that focus more on the initial stages of SUD showed that a significant proportion of children in Germany are affected by critical substance use by a parent.^6^

Estimates show that about 3 million children in Germany have lived in recent years with at least one parent who is addicted to legal and/or illegal substances.^5–7^ The majority of these, around 2.65 million children, lived in a household with parents with alcohol use disorder, and around 40.000 to 60.000 children have parents who used illegal substances. Additionally, there are behavioural disorders such as gambling.^7^ Other estimates showed that around 1.5 million - 2.7 million children were living with parents with a SUD (including tobacco use disorders).^8^ Around 690.00–1.26 million children were living in households where at least one adult had an alcohol use disorder, 88.000–160.000 in households where at least one adult had a disorder related to the use of illicit drugs (this study used DSM-IV diagnostic criteria to assess SUD related to tobacco, alcohol, cannabis, cocaine, or amphetamine).^8^ If risky alcohol consumption (not abuse or SUD) is considered, the numbers are even higher: Findings from another German study indicated that 22% of the parents with at least one underage child showed risky alcohol consumption.^9^ Given the average number of children of these parents, it can be estimated that up to 6.6 million children in Germany live with a parent with risky alcohol consumption.^9^ Current data from the German addiction support statistics show that around a quarter of the clients have at least one child of their own (outpatient: 27%, inpatient: 27%).^10^ However, it is not clear whether these individuals also live with their children or not.

### Consequences of Parental SUD and Effects on Parenting Behavior

Parental SUD represents a considerable social and economic burden for society.^5^,^11–13^ In addition, it has severe consequences for the users themselves (eg, health risks) as well as for the children who live with them. Physical and mental comorbidities, as well as psychosocial and social difficulties, are highly prevalent among parents with SUD.^14–19^ Parents’ SUD and its different consequences place numerous burdens on the affected families.^20^,^21^ For example, parental SUD poses a serious risk to the healthy development of their children.^22^ In particular, direct and indirect exposure to (il)legal substances (such as poor living conditions, less financial resources, child does not want to bring friends to his home/feels ashamed) can lead to socio-economic disadvantage, social exclusion, separation from parents, placement in out-of-home care, parentification, neglect of children, and more frequent family conflicts and domestic violence.^21^,^23–25^

Parents with SUD often show a lack of parenting skills, which can lead to dysfunctional, harmful, and/or traumatizing parenting behavior and an unstable parent-child bond.^21^ Children who were exposed to prenatal substance usage often suffer from long-term consequences: In addition to delayed cognitive and/or physical development, children of parents with SUD are a key risk group for developing a SUD themselves or other mental health problems, such as social behavior disorders, depression, or anxiety disorders. Around two-thirds of children who are exposed to SUD in their families develop a mental illness themselves or develop a SUD if they do not receive sufficient professional support.^5^,^6^,^26^,^27^ Children who live with parents with SUD are also disadvantaged in terms of their educational performance.^26^ Concerning alcohol, for example, there is evidence that the more alcohol consumed by adults living in a household, the poorer the (school) performance of children living with them.^24^,^26^ Studies have also shown that the lower performance of the children is clearly due to the psychopathology and psychiatric comorbidities of the parents.^28^

Given the multiple problems parents with SUD and their families are facing, the parental role and associated responsibilities often represent considerable burdens and excessive demands. In turn, the risks for children’s social and emotional functioning can be reduced by constant contact with parents without SUD or other significant adults (eg, teachers, friends, other relatives without SUD).^25^,^26^ Contact with parents who now live abstinent also helps to limit the adverse health and social effects on the children.^26^ Nevertheless, the affected parents urgently need support in coping with their potentially adverse, harmful, and/or traumatizing parenting behavior and its effects on their children.^29^

### SUD in Parents: Treatment and Treatment Approaches

On an international level, different treatment options and programmes are aimed explicitly at parents with SUD and have proven to be effective,^23^ for example, the “Parents Under Pressure” (PuP) program for parents with substance misuse problems.^30^ Here, the focus is on topics such as building or improving the parent-child bond; parents learning how to organize child raising and regulate their emotions.^30^ Another study from the USA examined the implementation of a family-centered substance use treatment for pregnant and postpartum people. The authors found that meeting needs on topics such as parenting skills and attitudes is essential in treating parents with SUD.^31^ Other programs that have proven to be effective, specifically focus on the parent-child bond, for example, the “Mothers and Toddlers Program (MTP)” or “Attachment and Biobehavioral Catch-up (ABC)”.^23^ Furthermore, there are evidence-based and effective behavioral and attachment-oriented interventions in the US that have been developed specifically for the needs of parents with SUD. The MATRIX outpatient program, for example, is explicitly aimed at methamphetamine using people and their families. The program focuses primarily on teaching parenting skills, psychoeducation, sexuality, and pregnancy in the context of crystal meth consumption.^23^ However, the above-mentioned programs, their content, and the associated needs of parents with SUD cannot be transferred one-to-one to the inpatient German addiction treatment system. For example, PuP, MTP, and ABC are home-based and not designed for the inpatient context. In Germany, there is hardly any evidence-based intervention that is explicitly aimed at helping parents with SUD in inpatient treatment.

The topics of parenting and parenthood have so far played a relatively minor role in inpatient rehabilitative addiction treatment in Germany. Although the German addiction treatment system is relatively extensive and well-established, the needs and requirements of parents with SUD tend to take a back seat in therapeutic and inpatient rehabilitative treatment. Even though it is partially possible to take children into medical rehabilitation, they are not treated, and mostly do not take part in therapies. Only some clinics have developed non-evidence-based individual programs to include children in their parents’ rehabilitation treatment, but the focus is still on adults.^32^

However, some programs and interventions in *outpatient treatment* have already been evaluated in Germany. These include, for example, programs like Mothers Support Training (in German: Mütter-Unterstützungsprogramm - MUT!), the SHIFT parent training (“Addiction Help and Family Training”; in German: “Suchthilfe und Familientraining”), as well as SHIFT Plus.^23^,^33^ These programs target specific groups of parents and do not take place in an inpatient rehabilitative context, which is why the needs of the parents may differ, and they cannot be fully transferred to the clinical rehabilitative context.

### Required Research and Aim of the Study

To sum up, in recent years, around 3 million children in Germany have lived with at least one parent who is addicted to legal and/or illegal substances. Parental substance use disorder places a considerable social and economic burden on society. It also has serious health consequences for the parents themselves and their children. At the same time, there are no addiction rehabilitation programmes that explicitly include services for parents with SUD, and evidence on the needs for parent-related rehabilitation treatment and the associated challenges is not yet available in Germany. This represents a significant gap in the literature as well as in the rehabilitative care of this vulnerable target group. Furthermore, results from other countries are difficult to transfer due to the specific nature of the German addiction treatment system.^29–31^ Therefore, the study aims to contribute to closing the literature gap with the purpose of exploring the parenting-related needs of parents with SUD in inpatient rehabilitation from an expert perspective in Germany. The study also seeks to uncover both structural and general challenges concerning the topic of “parenting and SUD” that need to be considered in inpatient rehabilitation. By doing so, optimization potential for inpatient rehabilitation for parents with SUD can be derived, and inpatient treatment can be improved. The study focuses on the following research questions:

- What parenting-related needs of parents with SUD for inpatient rehabilitative treatment can be identified from the experts’ perspective?
- What (structural) challenges for inpatient rehabilitative treatment concerning the topic of “parenting” can be identified from the experts’ perspective?

## Material and Methods

### Study Background

This study is part of the project “KontextSucht” (English: “AddictionContext”) running from 2021 to 2026, funded by the German Federal Ministry of Labour and Social Affairs (grant number 662S0053X1-1).^34^ The project is led by the German pension insurance of Central Germany (German: Deutsche Rentenversicherung Mitteldeutschland). The main objective is to develop, implement, and evaluate an intervention (German: KSI – KontextSucht Intervention, English: AddictionContext Intervention) for the target group of parents with SUD and children between the ages of 0 and 14 years that aims to improve parenting skills, parent-child bond, as well as parenting behavior. Ultimately, the aim is to reduce parenting problems and associated social and health consequences. The intervention should be able to be rolled out to comparable rehabilitation clinics in Germany afterwards. The evaluation of the intervention is led and carried out by the Technical University of Munich (TUM), TUM School of Medicine and Health, Chair of Social Determinants of Health. As part of a parallel mixed-methods study, we explored the general needs and challenges of parents with SUD as well as structural issues from the expert perspective to improve rehabilitative care for this vulnerable target group.^34^ For this purpose, we conducted interviews with experts from different rehabilitation clinics in Germany. This study presents the results of the expert interviews.

### Sampling

We used purposive sampling to obtain an information-rich sample of experts working in inpatient rehabilitation with parents with SUD.^35^ We, therefore, purposefully selected experts who were expected to contribute a great deal to answering the research questions.^36^

We interviewed four experts at two intervention clinics and nine at five non-intervention clinics that did not execute the KSI. The final sample consisted of 13 experts from different professions (including medical doctors, psychologists, psychotherapists, social therapists, dieticians, and curative education nurses). A total of nine women and four men participated in the study. At the time of the interviews, the interviewees were 40 years old on average (min. 28 years, max. 65 years). Sample details are shown in Table 1.

**Table 1:** Sample Selection

Sample	Intervention Clinics (n=2)	Non-Intervention Clinics (n=5)
Number of participants	n=4	n=9
Sex	Women n=4	Women n=5; men n=4
Average age	32,8 (28–44 years)	47,2 (30–65 years)
Professions	Psychologist, social therapist, dietician, curative education nurse	Medical doctors, psychotherapists

### Data Collection

The responsible research associate (AS) carried out the empirical data collection. We used semi-structured expert interviews as the data collection method. Expert interviews enable the collection of specialist knowledge about the research field, which is to be distinguished from everyday knowledge and common-sense knowledge.^37^,^38^ We assume that the experts from the direct care of persons with SUD have knowledge (eg, about contextual factors, the healthcare system, treatment options, etc.) that remains hidden from the lay/patient perspective. Expert knowledge is particularly valuable for improving the rehabilitative care of parents with SUD.

Findings from the international literature, as well as knowledge about the current status of rehabilitative care for parents with SUD in Germany, were used to inform and construct the interview guide. We further followed the specific steps for creating an interview guide by Helfferich.^39^ The interview guide was pilot-tested before conducting the interviews. Twelve interviews took place face-to-face at the clinics in an undisturbed environment, whereas one interview was conducted online via Zoom.^40^ All interviews were conducted between March and September 2023. They were audio-recorded with the participants’ permission, transcribed verbatim, and fully pseudonymized by a professional transcription office. The interviews were conducted and analyzed in German. The interview excerpts presented in the results section were translated into English by the authors.

### Data Analysis

The data analysis was based on Mayring’s structured content analysis.^41^ This method and its various forms are widely used in different research areas, including health and health services, as well as rehabilitation research.^42^ It offers a structured and rule-based approach to analyze qualitative interview data. The analytical view on the data was largely a realist ontological perspective. That means we considered the participants’ reports to be straightforward representations of their perceptions and experiences as they occurred in an “objective” reality.^43^ A content-structured form of deductive-inductive content analysis was used to analyze the expert interviews.^44^,^45^ Therefore, we developed an initial coding system (deductive part of the analysis) to create main categories and separated the interview material into different thematic sections. Further, subcategories were created during the inductive coding steps of the analysis. We used MAXQDA Analytics Pro 2022 to organize and store the data. Different criteria for measuring rigor were included in our study.^46^,^47^ Two researchers (AS, LH) were involved in coding the data to achieve reliability, intersubjective comprehensibility, and transparency. We discussed any inconsistencies that arose during the coding process within the research team.^47^ We also hold regular meetings to discuss and refine our analysis. This is a common and recommended way to increase the quality of qualitative analyses.^47^ We further followed the internationally consolidated criteria for reporting qualitative research (COREQ).^48^,^49^

### Ethics

The study was carried out following the principles of the Declaration of Helsinki and the standards of good scientific practice. Our study was approved by the Ethical Review Committee of the Department of Sport and Health Sciences of the TUM (approval no: 2022–624-S-KH). All participants were extensively informed about the project and the handling of the collected data by the responsible research associate (AS). Every participant provided written informed consent, including the publication of anonymized responses/direct quotes, before participating in the study.

## Results

The analysis of the expert interviews revealed detailed parenting-related needs of parents with SUD concerning inpatient rehabilitative treatment as well as associated (structural) challenges.

### Parenting-Related Needs

The interviewed experts all agreed that patients with SUD with children differ strongly from patients without children. They all start their therapy with numerous challenges in relation to their disorder and their role as a parent that need to be addressed. For example, the interviewed experts described the patients as less stress-resistant, which makes it much more difficult for them to take care of their children. They experience problems structuring daily (family) life (eg, getting the children up, preparing breakfast, taking them to kindergarten, etc.) and show reduced sensitivity in relation to recognizing and understanding the children’s needs, which significantly impairs age-appropriate support and caring for them.

It already starts with the daily routine, that the basics, making food, preparing meals, sending them to school, don’t work; they have no idea how to organize their free time with their children, apart from just handing them a tablet. And very often they don’t prepare food for their children, do they?” (Psychiatrist and senior physician, female, non-intervention clinic)

But then there is also the issue of adequately perceiving and interpreting the child’s needs because it is often difficult to recognise your own [the parents’] needs. The addiction compensated for this. (Psychotherapist, female, non-intervention clinic)

What does my child need? What are my child’s needs? Yes, and to recognize that, yes, especially when they’re being stubborn, they often take it personally and think, ‘Oh dear, now you’re being stubborn, you want to annoy me,’ but then they don’t have any sense of: Okay, what has just triggered the tantrum? Why are you angry? Why did that happen?” (Parent-child group therapist, female, intervention clinic)

The same expert described some of the “basics” of parenting, which are often missing, as follows:

Well, I think all sorts of things. So (.), yes, how can I make my child stay in bed at night? How can I make my child stay at the dinner table so that it does not get up? How can I regulate myself so that I do not freak out? So really (.) quite (.) wide-ranging. (Parent-child group therapist, female, intervention clinic)

The reduced sensitivity towards the children and difficulties in recognising their needs are well expressed in the following quote:

So, the most obvious thing is probably the general way of dealing with emotions, with crises, (.) with sensitivity towards the child, being attentive. These topics have probably been neglected in the past, depending on how old the child is. (Psychologist and therapist, female, intervention clinic)

Further, a low level of the ability to tolerate frustration and difficulties in self-regulating emotions makes dealing with the children even more difficult for the parents.

And then there are also patients who still have significant deficits, whether in terms of their ability to self-regulate or a lot of work [therapeutic work] needs to be done on controlling impulses. (Psychotherapist, female, non-intervention clinic)

It often happens because they haven’t learnt this from their family. Emotional regulation, they lack (.) the understanding that they are also a role model for their children, and that the children look at what they do, etc. (…). (Psychological psychotherapist in training, female, non-intervention clinic)

According to the expert interviewees, the parents generally lack parenting skills, as they are unable to adequately care for the child due to their SUD and associated comorbidities. For example, they have problems giving emotional support, showing love, and offering age-appropriate help. They also have a hard time dealing with daily tasks. This is also observed in the clinic (with the option to bring accompanying children) by the experts, for example, as the parents do not regularly take part in meals with their children during their inpatient stay, and have reduced or no knowledge of how they can spend their free time with their children in the clinic in an age-appropriate way.

If I look specifically at my area of work, I see a huge deficit in the fact that parents don’t go out to eat with their children regularly. The background to this is that I personally still work in the kitchen. That means I’m there for breakfast, lunch, and dinner. It’s really noticeable that the children don’t go to breakfast with their parents at all. At lunchtime, there are one or two patients who don’t come to lunch, and they don’t come to dinner either. (Dietician, female, intervention clinic)

This results in various needs, but also requirements that parents with SUD take with them into their rehabilitation stay. One of the experts summarised this very clearly as a “commitment to parenthood”:

Well, the desire (.) to say: ‘I am now fully committed to my child. There’s nothing else to distract me. The drug no longer controls me, the substance, but I can decide for myself what my life should look like with my children.’ So, this ‘I’m building something of my own again’, that’s a huge need. (Psychologist and therapist, female, intervention clinic)

The experts further described the parents’ desire for attachment and security as a significant and substantial parental need. They stated that without feeling attachment security themselves, it is difficult or almost impossible to pass it on to the children. The experts also described a parental need for a sense of community, role models, as well as a need for rest and relief.

So, I think the most important need is for attachment, for security, right? Somehow. And many rehabilitants say that too, so: ‘I feel safe here [in the clinic]. I don’t want to leave here. I’m fine here, well here. My child is fine here.’ The children, yes, are happy. Sure, the parents are somehow clear-headed again. So, I think there’s this need for security and for someone to show me what life can be like. (Parent-child group therapist, female, intervention clinic)

This statement underlines that parents need a place where they know they and their children are safe and well looked after - a so-called “safe place”. The clinic basically offers the conditions to fulfill this requirement, and in this context, the parents can heal, rest, and work on their recovery process with the help of therapeutic work to ultimately be able to take better care of their children. This safe place is also essential for the children to calm down and (re-)build connection and a feeling of security with their parents. The following quote shows that there is still room for improvement in terms of implementation:

And it’s the same with the little ones, depending on whether they were really with their parents in the first place, or whether they were also partly housed away from home, as they say, this feeling of community. So, I always link breakfast, lunch, and dinner as a family with community. You sit together at the table. Sometimes it’s the only meal where you can talk and eat together. I sometimes miss this sense of community here. It has to be done very quickly, and I pick up my child shortly beforehand, and then eat quickly and drop the child off again. Mhm, I think that’s difficult. I think that’s something where they, the parents, have to work a lot more with themselves at that moment or work on the children and themselves” (Dietician, female, intervention clinic)

To sum up, the experts described a lack of topics that should be included in therapy and options where parents with SUD can work on their parenting-related tasks during their inpatient stay. These are summarised in Table 2.

**Table 2:** Parenting and “Adulthood”-Related Topics and Options That are Currently Missing in Inpatient Rehabilitative Care

Issues that are Related Explicitly to Parenthood	“Parental Self-Management Skills”
Organising daily life with a child/children and taking over responsibility for them	Expressing and regulating emotions
Shaping the relationship with the children (eg, building a stable parent-child bond, dealing with conflicts)	Space for the biographical work of the parents
Providing support on “how to interact with children”	Assistance with administrative matters (eg, youth welfare office)
Parental role	Support for parents and children after rehabilitation (eg, outpatient services)
Programs about the general development of children (including behavioral issues and the effects of substance use disorders on children)	

### (Structural) Challenges in Connection with the Topic of “Parenting and SUD”

In addition to the needs of parents with SUD, there are also challenges that need to be addressed in order to adequately therapeutically address the topic of ‘parenting’ in inpatient rehabilitation in the future.

Overall, the interviewed experts reported challenges regarding staff and a lack of financial resources to offer additional but necessary services. Currently, it is not possible to meet the needs of parents with SUD concerning services about parenting and parenthood - ie, beyond the programs offered as standard therapy in inpatient rehabilitation. Another problem that comes along with low financial resources is a lack of materials (eg, toys, craft and sports materials, furniture for children, and sanitary products) to adequately care for children. The following quote further underlines the low priority of including the above-mentioned issues in rehabilitation, as it is described as too expensive:

Yes, one thing we briefly mentioned earlier was that I don’t know much about it, but as I understand it, in terms of costs, it’s not something where providers say: ‘Wow, it’s worth it. We’ll definitely do that.’ Rather, it’s something that, let’s say, must be a matter close to someone’s heart rather than something that makes much economic sense, right? (.) It’s just work-intensive. And therefore, also expensive. (Senior physician and specialised doctor for psychiatry and psychotherapy, male, non-intervention clinic)

The interviewees described a further challenge concerning cooperation with youth welfare offices, daycare centers, and schools. There is a lack of communication and links or connections between clinics and local authorities. The experts would specifically like to see greater involvement of various local authorities and institutions and, as a result, better cooperation with institutions outside the clinic, such as daycare centers, schools, and youth welfare offices.

Maybe better cooperation with the youth welfare office, right? It’s always like that, I’ll say ominously somewhere in the background, but there’s no real contact. It would perhaps make sense for all sides if there were better contact options, even if it’s only by telephone, right?. (Senior physician and specialised doctor for psychiatry and psychotherapy, male, non-intervention clinic)

The option of placing the children in schools or kindergartens during their parents’ stay also influences their willingness to attend inpatient therapy. The experts describe that parents are sometimes not willing to be separated from their children for a long period of up to 24 weeks and to be “alone” in the rehabilitation clinic. However, if schools and other care facilities are not available or only available to a limited extent, this has a significant impact on parents’ utilization of rehabilitative services, as it can be seen in the following quote:

We have to organize a host school. And this is urgent for us in the region, I don’t know if you have this locally, that, of course, the schools are simply bursting (…), and we then have the difficulty of accommodating host pupils because they say ‘we’re full’, right? And that means that if you have a family with a schoolchild, you can say: ‘Yes, I can’t get the child into school now.’ But we have mandatory schooling, and they say: ‘Yes, I won’t do it without my child’, right? That can sometimes be a hurdle. (Head of the family division, male, non-intervention clinic)

In terms of structural barriers, the experts made clear that the varying duration of treatment depending on the insurance provider and indication is also a problem for the treatment of parents with SUD. For example, the duration of rehabilitation differs depending on the substance used (12 weeks for alcohol and up to 24 weeks for drugs). A shorter duration of therapy makes it even more challenging to integrate therapy programs related to parenting alongside the already standard programs for the patients.

Social Security Act 6 is one thing, and the duration of therapy is another. So, for many patients, the different cost commitments, which sometimes range from a few weeks to 24 weeks, and many don’t need that much at all, and others, who perhaps need more, don’t get any due to whatever formal circumstances. Yes, then you have to reduce it to what we can actually provide here in the period of time. (Senior physician, female, non-intervention clinic)

With regard to therapy content and the organization of this, the experts found it difficult to “burden” their patients, in addition to their already full therapy schedule, with additional therapy topics, especially parenting-related topics. In this context, the issue arose that patients first had to concentrate fully on themselves and their SUD and work on this before they were ready to focus on their social environment and family-related issues. The topic of parenting is very complex and entails extensive demands. There is often a lack of time to support the family as a whole or parents and children as required.

Also spending time with, I don’t know, spending time together also with a therapist as therapeutic play. (.) so that my therapist also accompanies, perhaps, several patients at the same time or just one [family]. There’s not much time for that. (Psychological psychotherapist in training, female, non-intervention clinic)

## Discussion

### Summary of the Results

Substance use disorders can often harm not only the person directly affected but also their family and children. Although the negative health, social, and environmental consequences on parents with SUD and their children and families are widely known, they are not systematically addressed in German inpatient rehabilitation treatment. The results of our expert interviews reveal that there is a strong need for rehabilitation measures that not only focus on the SUD itself but also take the topic of parenthood with its various specific issues explicitly into account. This would include, for example, different parenting-related topics such as organizing daily life with a child or children, shaping the relationship with the children, programs about the general development of children, expressing and regulating emotions, and assistance with official/administrative matters. Overall, the experts describe a lack of opportunities where parents with SUD can work on their parenting-related tasks during their inpatient stay.

Even more, there are various structural issues that need to be addressed. For instance, rehabilitation clinics in Germany currently need to create programs for parents and children on their own, more or less at their own expense, and over and above their regularly funded therapeutic services, if they want to address parents’ and children’s needs. Currently, no services specifically for parenting-related issues are considered or officially sponsored by standard rehabilitative care (eg, by pension or health insurance). To overcome these issues, it is necessary, for example, to call for policy changes concerning insurance coverage (eg, expanding insurance coverage by including parenting-related matters).

In addition, we were also able to identify several barriers from an expert perspective that currently hinder the implementation of the missing services. These include, first and foremost, a lack of staff, time, and financial resources, which might be overcome by changing the allocation of funds. Further, the experts would specifically wish for greater involvement of various local authorities and institutions and, as a result, better cooperation and communication with institutions outside the clinics, such as kindergartens, schools, and youth welfare offices.

### Comparison with Previous Research and Other Programs

In Germany, our study is one of the first to provide findings on the needs and challenges of parents with SUD in inpatient rehabilitation treatment from an expert’s perspective. Compared to previous (international) studies on the consequences of SUD on families, we see that these largely align with the identified needs. Our results show that affected parents need, for example, offers for structuring the day with children and shaping the relationship with the children. It is well known that parents with SUD are often challenged by parenting skills, which can lead to dysfunctional parenting behavior and impaired parent-child relationships.^21^ It can be assumed that if the needs of the affected parents are met by providing appropriate support during their rehabilitation stay, this will have a positive effect not only on the parents but also on the children and the entire family. For example, as part of our results, programs on “how to interact with children”, parental roles, and the general development of children may reduce adverse effects on both the children and the parents. Other topics, like expressing and regulating emotions and space for parents’ biographical work, will likely improve parenting behavior. Children who live with parents with SUD are also disadvantaged in terms of their educational performance.^26^ Assistance with administrative matters (eg, youth welfare office) and support for parents and children after rehabilitation (eg, outpatient services) can also help compensate for some of these difficulties.

Evaluated programs from the USA and UK that specifically aim at meeting the needs of parents with SUD and have proven effective in improving parenting skills often include topics such as parental roles and regulating emotions.^23^ Using a randomized controlled trial, a team of researchers from the UK evaluated the “Parents Under Pressure” (PuP) program for parents with substance misuse problems.^30^ Here, the focus is clearly on topics such as building or improving the parent-child bond, parents learning how to organize child raising, and, again, how to regulate their emotions. In addition, relapse strategies are developed.^30^ Although these programs take place in other settings (outpatient or home-based), they largely cover topics that may meet the needs of parents with SUD, which we have also identified in our study. A qualitative study from the US, which examined the implementation of a family-centered substance use treatment for pregnant and postpartum people, also concluded that topics such as parenting skills and attitudes are essential in the treatment of parents with SUD.^31^ However, the programs mentioned above, their content, and the associated needs of parents with SUD cannot be easily transferred to the very complex German treatment system. There are numerous outpatient (eg, addiction counselling) and inpatient (acute care, rehabilitation) services, post-treatment care, and prevention services. Everything is primarily financed and organised by the state according to established structures.^10^

In comparison, the addiction treatment system in the United States, for example, is much more fragmented, and services are less well coordinated. In addition, treatment costs are significantly higher. Therefore, services from this more fragmented care system are challenging to transfer to the structured German system. Structural adjustments are consequently needed to integrate rehabilitation services for parents with SUD.

However, in Germany, there are hardly any evaluated interventions that specifically aim at meeting the needs of parents with SUD in inpatient rehabilitative care. Nevertheless, there are a few outpatient programs, such as the Mothers Support Training (in German: Mütter-Unterstützungsprogramm - MUT!) or the SHIFT parent training (‘Addiction Help and Family Training’; in German: “Suchthilfe und Familientraining”), which also aim to improve parenting and educational skills.^23^ However, these programs target particular groups (eg, SHIFT is aimed at methamphetamine-addicted parents), and both parent training courses take place in outpatient settings, which is why the needs of the parents in inpatient settings may differ, and they cannot be fully transferred.

Despite the similarities with US and UK programs, our results once again provide new insights into the needs and challenges to improve rehabilitative care for parents with SUD in Germany (eg, including topics like shaping the relationship with the children, programs about the general development of children, assistance with official/administrative matters or structural issues like a greater involvement of local authorities and cooperation with institutions such as kindergartens, schools, and youth welfare offices). Many programs in the US addiction support system are outpatient or home-based. In contrast, our study focused on the inpatient, rehabilitative context in order to reach and comprehensively support parents during their relatively long stay in the clinic. Our results from the experts’ perspectives were thus able to show very specifically what needs parents with SUD have, especially in an inpatient, rehabilitative setting, and what structural barriers and challenges exist.

The results of the present study extend the existing (international) evidence of the needs and hurdles of SUD parents in inpatient care and contribute to the development of new interventions (such as the KSI) to support parents with SUD in inpatient care to improve health, quality of life, and functionality of the affected families in the future.

## Strengths and Limitations of the Study

A few limitations of the current study need to be acknowledged. It can be assumed that the direct recruitment of experts in the clinics, especially in the intervention clinics, resulted in only highly motivated participants being interviewed, who may have assigned a higher priority to the topic from the outset. However, as we found various similarities with other studies from the USA and the UK, we can assume that this selection bias had a rather minor influence on our results. When selecting the experts, we also took special care to achieve a diverse sample to obtain different views on the research topic. We further found consistent results across different professions, particularly from the non-intervention clinics compared to the intervention clinics. Still, most participants were female, which may also limit our findings. In future studies, a more balanced sample of different genders, geographical regions, and clinic types is needed to further increase the generalisability of the results.

The second limitation relates to the expert perspective itself, which is important and new in this context, but this is only one view of the topic of interest. The expert views may be limited in reflecting the direct needs of parents with SUD. To investigate this further and comprehensively, it is necessary and essential to also examine the perspective of parents with SUD. In addition to structural hurdles and challenges, which can be better analysed from an expert’s perspective, the parents’ perspective offers even more detailed insights into their specific wishes and needs. More research needs to be done regarding this point. Last, we used a relatively small sample of participants. In larger-scale studies, with more clinical and interdisciplinary experts, the results could be further validated. However, this limitation has a relatively small influence on the results since we took care in selecting an appropriate sample size to interview an adequate number of experts, as well as a multi-centric design in order to be able to draw reliable conclusions.^50^ The size of the sample is not the most important factor in qualitative studies, but it is about how rich in information the collected data is.^50^

The limitations described above stand in contrast to the added value of this study in the field of research on the rehabilitative care of parents with SUD, as the present results provide for the first time an interdisciplinary overview of the needs, challenges, and possibilities for improvement of the quality and structure of inpatient rehabilitative care of parents with SUD from the perspective of various experts in Germany.

## Conclusion

The possible adverse effects of parents with SUD on their children have been extensively documented. The resulting need for care in an inpatient setting is justified by the lack of concepts to date. Even though there are several programs from other countries, their content and their results cannot be transferred to the unique and complex German addiction treatment system. The results presented here emphasize the concrete need for an evidence-based intervention for family-focused addiction treatment. In this way, care and the resulting health, quality of life, and functionality of the affected families can be improved in the future. The abstinence of the affected parents can be ensured in the medium term and thus also reduce the addiction-related burden and adverse health effects on the children. In future studies, it is important to also include parents with SUD to further strengthen and enrich our findings.

## Abbreviations

SUD, substance use disorder; US, United States.
